# Pneumocystis Infection in Two Patients Treated with Both Immune Checkpoint Inhibitor and Corticoids

**DOI:** 10.4103/JIPO.JIPO_23_19

**Published:** 2020-02-10

**Authors:** Maroun Sadek, Angela Loizidou, Annie Drowart, Sigi Van den Wijngaert, Maria Gomez-Galdon, Sandrine Aspeslagh

**Affiliations:** 1 Department of Medical Oncology, Institut Jules Bordet, ULB, Brussels, Belgium; 2 Department of Internal Medicine, Institut Jules Bordet, ULB, Brussels, Belgium; 3 Department of Microbiology, University Laboratory LHUB-ULB, Brussels, Belgium; 4 Department of Anatomopathology, Institut Jules Bordet, ULB, Brussels, Belgium; 5 Department of Dermatology, Erasme Hospital, ULB, Brussels, Belgium; 6 Department of Medical Oncology, Erasme Hospital, ULB, Brussels, Belgium; 7 Department of Medical Oncology, UZ Brussel, VUB, Brussels, Belgium

**Keywords:** Immune-related adverse events, immunotherapy, ipilimumab, nivolumab, pembrolizumab, Pneumocystis pneumonia

## Abstract

The introduction of immune checkpoint inhibitor (ICI) targeting cytotoxic T-lymphocyte-associated antigen-4 and programmed cell death receptor 1 has dramatically improved clinical outcome for cancer patients. Nevertheless, this treatment can be associated with immune-related adverse events (irAEs) which sometimes need management with prolonged immune suppression. In order to analyze the risk of *Pneumocystis jiroveci* pneumonia (PJP) in this population, all PJP cases at our oncological hospital between 2004 and 2019 were searched. Only two cases were found in patients treated with ICI (480 patients received ICI during that period). The first was treated with both ipilimumab and nivolumab for metastatic melanoma and required long-term corticosteroids plus infliximab for immune-related colitis. The second received both pembrolizumab and brentuximab for Hodgkin's lymphoma and received corticosteroids for macrophage-activating syndrome. These two cases illustrate that PJP is rare but might be severe in the ICI population and should be differentiated from tumor progression or irAE.

## Introduction

Long-term corticoid usage induces immunosuppression, which may lead to opportunistic infections such as pneumocystis. Treatment with immune checkpoint inhibitor (ICI) may lead to immune-related adverse events (irAE) requiring long-term corticoids and therefore may be at risk of developing *Pneumocystis jiroveci* pneumonia (PJP).

We performed a retrospective study in our oncology hospital, during a period of 14 years (2005–2019): 1643 tests for polymerase chain reaction (PCR) and/or immunofluorescence for *P. jiroveci* (PJ) were performed and 35 were found positive. Six patients (one HIV and five lacking clinic of PJP) were excluded. Two patients received ICI; the majority (76%) underwent corticosteroid treatment (22/29) and three patients had fulminant presentation and died due to PJP (one had not been treated). The incidence of PJP in anti-programmed cell death (ligand) 1 receptor anti-PDL1-treated patients was very low: 0.42%: 2 out of 480 patients (anti-PD1: 2/279 and anti-PDL1: 0/201).

## Case 1

**Figure 1:**
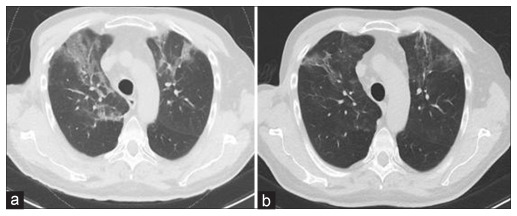
Imaging of PJP infection. Computed tomography scan of the thorax showing diffuse interstitial infiltrates bilaterally correlating with (a) Pneumocystis jiroveci pneumonia and (b) clearing of these infiltrates after 2 weeks of trimethoprim/sulfamethoxazole. PJP: Pneumocystis jiroveci pneumonia.

**Figure 2:**
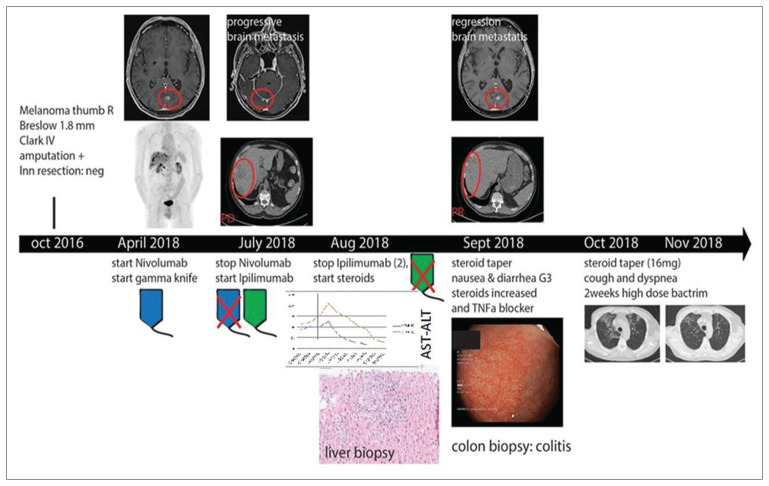
Timeline case 1. This figure illustrates tumor, colitis, hepatitis, and PJP evolution for case 1. PJP: Pneumocystis jiroveci pneumonia, TNF-α: Tumor necrosis factor-alpha.

The first case is a 68-year-old patient with a melanoma of the right thumb (Breslow 1.8 mm), treated by amputation; pT2aN1M0. Two years after the initial diagnosis, metastatic disease appeared in the liver, lung, and brain. Hereupon, nivolumab was started, and the brain lesions were managed by gamma-knife radiosurgery. After seven cycles, the patient progressed and nivolumab was switched to ipilimumab (anti-cytotoxic T-lymphocyte-associated antigen 4). After two cycles, the patient presented with fever and Grade III aspartate aminotransferase and alanine aminotransferase (NCI CTCAE v5), and liver biopsy revealed immune-related hepatitis. Ipilimumab was stopped and methylprednisolone was started at a dose of 1 mg/kg with marked improvement.[1] Three weeks later (while on 8 mg of methylprednisolone), he experienced diarrhea Grade II and was diagnosed with immune-related colitis which was managed by increasing methylprednisolone to 125 mg; however, because of worsening diarrhea, infliximab (anti-tumor necrosis factor-alpha [TNFα]) was instored at 5 mg/kg upon which colitis quickly resolved. Seven weeks later, he was readmitted for fever, cough, and mild dyspnea (while on 16 mg of methylprednisolone for 10 weeks). Physical examination revealed bilateral basal crackles, without tachycardia; oxygen saturation was 93% and partial pressure of oxygen was 65 mmHg at room air. Blood analysis showed a C-reactive protein of 72 mg/L (normal range <10 mg/L), lactate dehydrogenase slightly elevated (460 UI/L, normal range: 135–225 UI/L), the absolute lymphocyte count (ALC) was 720/mL, and CD4 T cells were 266.4/mL (normal range: 410–1590/mL). Thorax computed tomography (CT) scan showed diffuse, bilateral, and interstitial infiltrates [Figure 1]. Bronchoalveolar lavage (BAL) was positive for PJ (confirmed by PCR), and the patient was treated with 1600 mg sulfamethoxazole/320 mg trimethoprim (four times daily) intravenously (IV) for 14 days with fast clinical remission [Figure 2].

## Case 2

The second case was a female who developed Hodgkin's lymphoma at the age of 8 years treated by surgery, chemotherapy, and radiotherapy. Seven years later at the age of 15, she was again treated with chemotherapy due to mediastinal relapse. Sixteen years later, at the age of 24, she had a new relapse and was treated with three cycles of rituximab, dexamethasone, cytarabine, and cisplatin. Upon progression, this was switched to bendamustine–brentuximab under which after 20 days, she developed a macrophage-activating syndrome necessitating a single dose of etoposide and prolonged corticosteroids. She presented a *Mycobacterium abscessus* bacteremia, respiratory syncytial viral infection, and tracheo-bronchial necrotic aspergillosis (all 12 days after the beginning of corticosteroids). As Hodgkin's disease also relapsed, pembrolizumab (anti-programmed cell death receptor 1) was started. On the 2^nd^ day following the 6^th^ cycle of immunotherapy, she presented with fever up to 39.8°C with chills, headache, nausea, vomiting, and productive cough without dyspnea. Blood test confirmed chronic severe lymphopenia (ALC 210/mL), and she was still on 8-mg methylprednisone (started 6 months earlier at a dose of 16 mg and tapered to 8 mg for 4 months). Chest CT scan showed diffuse, bilateral, and interstitial infiltrates compatible with PJP, and immunofluorescence for PJ on BAL was positive although she was on prophylaxis with sulfamethoxazole 800 mg/trimethoprim 160 mg three times per week for at least 16 years (which she had stopped 3 weeks earlier due to intolerance). She was treated with 1600-mg sulfamethoxazole and 320-mg trimethoprim IV for 10 days followed by oral dapsone 100 mg once daily and trimethoprim 300 mg twice daily for another 11 days, Hereupon, she had a satisfying clinical and imaging response.

## Discussion

Here, we report two cases of PJP in ICI-treated patients with prolonged corticoid therapy. One of them also received anti-TNF-α. Both patients had lung noduli related to the underlying disease, nevertheless PJP diagnosis was quite rapidly made as in both cases the patients presented with fever and dyspnea, making tumor progression and checkpoint inhibitor-related pneumonitis (CIP) less likely.

Pneumocystis pneumonia is an opportunistic fungal infection caused by PJ, formerly designated *Pneumocystis carinii*.[2] Normally, colonization by pneumocystis is transient in immunocompetent individuals[3] and may become more persistent in HIV-infected patients.[4] PJP is an important cause of severe pneumonia in immunocompromised patients with cancer, organ transplant recipients, or those receiving immunosuppressant medications.[2] In literature, five cases of PJP due to irAE-related immunosuppression have already been reported[5–7] [Table 1]. Other factors that predispose to PJP are CD4 T cells <200 cells/ml or 20% of ALC and possibly *Cytomegalovirus* infection.[8]

**Table 1:** Main characteristics of the reported cases of immune-related Pneumocystis jiroveci pneumonia during immunosuppression for immune-related adverse events

**Cases**	**Age (years)/sex**	**Diagnosis**	**First-line treatment**	**Second-/third-line treatment**	**Immune-related adverse events**	**Opportunistic infection**	**Treatment**	**Outcome**
Schwarz M et al., 2019	79/male	NSCLC	Carboplatin-gemcitabine	Nivolumab	Pneumonitis treated by steroids	PJP	Piperacillin-tazobactam trimethoprim/sulfamethoxazole steroids	Death
Schwarz M et al., 2019	53/male	NSCLC	Cisplatin- vinorelbine followed by a right upper lobectomy	Nivolumab combined with radiotherapy	Pneumonitis treated by steroids	PJP CMV	Trimethoprim/sulfamethoxazole, steroids, broad-spectrum antibiotics, ganciclovir	Death
Arriola E et al., 2015	69/female	Melanoma	Surgical resection and lymphadenectomy	Dacarbazine and then ipilimumab	Colitis treated by steroids and infliximab	PJP	Trimethoprim/sulfamethoxazole	Recovery
Arriola E et al., 2015	63/female	Melanoma	Wide local excision and axillary block dissection	Ipilimumab	Colitis and capillary leak syndrome treated by steroids	PJP	Trimethoprim/sulfamethoxazole	Recovery
Slevin F et al., 2016	52/female	Melanoma	Vemurafenib	Ipilimumab	Ileitis and pancolitis treated by high- dose corticosteroids and infliximab	PJP	Piperacillin-tazobactam clarithromycin trimethoprim/sulfamethoxazole steroids	Recovery

PJP: *Pneumocystis jiroveci* pneumonia, CMV: *Cytomegalovirus*, NSCLC: Non-small cell lung cancer

Among the immunosuppressive drugs that predispose to PJP, corticosteroids seem to be the most widely culpable, especially with a median dose of 30 mg/day prednisone for 12 weeks (or 20 mg/day for at least 2 weeks).[8] In these cases, the American Society of Clinical Oncology (ASCO) recommends PJ prophylaxis as the risk of developing PJP is >3.5%.[9,10] Corticosteroids limit the number of alveolar macrophages (AMs) which are responsible for the clearance of PJ. This reduction is more severe when additional immunosuppression such as anti-TNF-α is used and/or lymphopenia is present as was the case for our patients. Interestingly, cessation of immunosuppression leads to rapid rebound of AM and should therefore be incorporated in the treatment of PJP.[11]

Onco/hematological patients who receive checkpoint may develop irAE, which sometimes requires long-term corticoids possibly combined with another immunosuppressive treatment. A recent abstract which was discussed at the ASCO 2019 analyzed PJP in CIP and found that 6 out of 23 CIP patients had a PCR positivity for PJP; interestingly, the total number of lymphocytes was significantly lower in the patients developing PJP and there was a trend of receiving higher and longer doses of steroids.[12] Our database was not detailed enough to look for PJP occurrence in the several different irAEs and our two cases did not have CIP. Nevertheless, both our patients also presented with low lymphocyte count albeit >200 CD4 T cells, which is often used as a cutoff. Unfortunately, the lymphocyte or CD4 T cell count in the five published PJP cases in irAE patients was not mentioned, so there is not enough evidence to set a clear lymphocyte or CD4 T cell threshold about when to start PJP prophylaxis in irAE patients. Nevertheless, as PJP might be severe, we would advise to start PJP prophylaxis when long-term corticoids (>1 month, >20 mg prednisone daily) and/or additional immunosuppression is required and/or severe lymphopenia is present. In addition, PJP should be a differential diagnosis in all patients suspected with CIP as PJP can mimic all radiological patterns.

### Declaration of patient consent

The authors certify that they have obtained all appropriate patient consent forms. In the form the patient(s) has/have given his/her/their consent for his/her/their images and other clinical information to be reported in the journal. The patients understand that their names and initials will not be published and due efforts will be made to conceal their identity, but anonymity cannot be guaranteed.
